# Distinct Features of Germinal Center Reactions in Macaques Infected by SIV or Vaccinated with a T-Dependent Model Antigen

**DOI:** 10.3390/v13020263

**Published:** 2021-02-09

**Authors:** Maria Trovato, Hany M. Ibrahim, Stephane Isnard, Roger Le Grand, Nathalie Bosquet, Gwenoline Borhis, Yolande Richard

**Affiliations:** 1 Institut Cochin, Université de Paris, INSERM, CNRS, 75014 Paris, France; maria.trovato@ibbc.cnr.it (M.T.); hanyibrahimeg@gmail.com (H.M.I.); stephane.isnard@mail.mcgill.ca (S.I.); 2 Université Paris-Saclay, INSERM, CEA, Center for Immunology of Viral, Auto-Immune, Hematological and Bacterial diseases (IMVA-HB/IDMIT), 92260 Fontenay-aux-Roses, France; roger.le-grand@cea.fr (R.L.G.); nathalie.bosquet@cea.fr (N.B.)

**Keywords:** B-cells, SIV, GC, T_FH_, CXCL10, CXCL13, BAFF, memory B-cells

## Abstract

B-cell follicles constitute large reservoirs of infectious HIV/SIV associated to follicular dendritic cells and infecting follicular helper (T_FH_) and regulatory (T_FR_) T-cells in germinal centers (GCs). Thus, follicular and GC B-cells are persistently exposed to viral antigens. Despite recent development of potent HIV immunogens, numerous questions are still open regarding GC reaction during early HIV/SIV infection. Here, we dissect the dynamics of B- and T-cells in GCs of macaques acutely infected by SIV (Group SIV^+^) or vaccinated with Tetanus Toxoid (Group TT), a T-dependent model antigen. Systemic inflammation and mobilization of antigen-presenting cells in inguinal lymph nodes and spleen are lower in Group TT than in Group SIV^+^. Despite spleen GC reaction of higher magnitude in Group SIV^+,^ the development of protective immunity could be limited by abnormal helper functions of T_FH_ massively polarized into T_FH1_-like cells, by inflammation-induced recruitment of fCD8 (either regulatory or cytotoxic) and by low numbers of T_FR_ limiting T_FH_/T_FR_ competition for high affinity B-cells. Increased GC B-cells apoptosis and accumulation of CD21^lo^ memory B-cells, unable to further participate to GC reaction, likely contribute to eliminate SIV-specific B-cells and decrease antibody affinity maturation. Surprisingly, functional GCs and potent TT-specific antibodies develop despite low levels of CXCL13.

## 1. Introduction

Various B-cell abnormalities associated with pathogenic HIV infection evidence several biases in the generation and function of virus-specific, post-germinal center (GC) effector B-cells [1,2]. In physiological settings, GC represents a unique place where B-cells stimulated by T-dependent (TD) antigens (Ags) undergo a complex and dynamic process of expansion and selection, allowing the generation of Ag-specific long-lived effectors: resting memory (RM, CD21^+^CD27^+^) B-cells and plasma blasts (PBs) [3,4,5]. Memory B-cells and PBs which ensure long-term protection against pathogens fail to cover this role during HIV/SIV infection, with progressive loss in HIV-specific and non-specific RM B-cells and HIV-specific antibodies (Abs) being globally inefficient in containing the viruses [1,2]. Indeed, if cross-neutralizing Abs with narrow breadth are frequently present during the first year of infection in HIV-infected individuals [6], most broadly neutralizing Abs (bNAbs) develop only after several years of infection and in a limited number of HIV-infected individuals [7]. A substantial fraction of bNAbs is produced by self/polyreactive B-cells which escape central or peripheral tolerance, as a consequence of chronic inflammation and exposure to viral Ags [8,9]. Nevertheless, these Abs frequently harbor high levels of somatic hyper-mutations in immunoglobulin (Ig) genes, arguing for their affinity maturation and selection in GC [7,10,11]. Studies of bNAbs have identified key vulnerability sites in the HIV-1 Envelope (Env) proteins [10,11], leading to the design of novel immunogens such as the SOSIP Trimers, biochemically stabilized native-like HIV-1 Env trimers that mimic the trimeric structure of HIV-1 spikes [12]. Recently, the BG505 SOSIP.664 trimers used as immunogen in optimized strategies of immunization succeeded in the development of robust Tier-2 NAbs in non-human primates (NHPs) [13,14]. In this animal model, neutralizing activities rapidly developed compared to neutralizing responses during HIV/SIV infection and were associated to high frequencies and quality of GC B-cells and follicular helper T-cells (T_FH_) following the first immunization. Beyond a possible broader recruitment of Env-specific B-cells, the absence of CD4 T-cell depletion might contribute to higher expansion of T_FH,_ with more potent helper activities. In contrast to macaques unimmunized or developing low titers of BG505-specific Tier-2 NAbs, those with high titers of NAbs showed significant protection from homologous SHIVBG505 challenge [15].

GCs are undoubtedly important dating sites where many cellular actors must dialogue appropriately to generate potent effector B-cells and a protective Ab response. Cognate interactions between GC B-cells and T_FH_ play a mandatory role in the positive selection and survival of B-cell clones expressing high affinity B-cell receptor (BCR) [5]. Present at elevated frequencies in GC during HIV/SIV infection, T_FH_ frequently exhibit impaired helper functions and cytokine production during the chronic phase of infection [16]. However, we recently showed that GC T_FH_ can exert efficient helper functions during the acute phase of SIV infection [17]. Infected at higher frequencies than other memory CD4^+^ (mCD4) T-cells, GC T_FH_ constitute a major reservoir of infectious virus in HIV-infected individuals, untreated or successfully treated by anti-retroviral therapy, as well as in elite controllers [18,19]. Follicular regulatory T-cells (T_FR_) expressing FOXP3 and endowed with immunosuppressive functions play a key role in regulating GC reactions through interactions with GC B-cells and T_FH_, preventing the development of autoimmunity [20]. Heightened T_FH_/T_FR_ ratio is associated with the development of self-reactive Abs during chronic SIV infection [21]. However, T_FR_ could also exert helper functions toward GC B-cells through IL10 provision [22]. Thus, the balance between immunosuppressive and helper functions of T_FR_ could vary from one setting to another and over time in a given setting [23]. More recently, T_FR_ were shown to be highly permissive to HIV infection, likely contributing to viral persistence in tissues [24]. Virions (infectious or not) released by T_FH_ or T_FR_ in GC are trapped into long-lived immune-complexes that decorate follicular dendritic cells (FDC) through binding to CD21 or FcγR. These immune-complexes establish an even greater reservoir of HIV RNA that is highly resistant to anti-retroviral therapies [25]. In physiological settings, only GC B-cells that can displace Ags from FDC-associated immune-complexes, establish cognate interactions with T_FH_ and receive appropriate cytokine signals from the GC microenvironment can differentiate into effector B-cells [5]. When chronic BCR occupancy and signaling occurs in GC B-cells with concomitant activation of Toll-like receptors (TLR) 7/9 and/or exposure to particular cytokines (IL21, IFNγ), this physiological process is disturbed and atypical (CD19^hi^CD21^lo^) memory B-cells are generated. These CD21^lo^ memory B-cells frequently express inhibitory receptors (FcRL, PD1, CD22, CD85j) and activation markers (CD80, CD86, HLA-DR) at high level, and are mostly T-bet^+^ and CD11c^+^ [26]. Although BCR signaling is altered in CD21^lo^ B-cells, their capacity of Ag-presenting cells (APCs) is preserved [27,28,29,30,31,32]. Largely absent in healthy individuals, a subpopulation of CD19^hi^CD21^lo^ memory B-cells, first described as tissue-like memory (TLM, CD21^lo^CD27^lo^) B-cells in HIV-infected individuals [33], is also present in other settings [26,34]. In a mouse model of plasmodium infection, this population was shown to be short-lived pre-plasma blast producing pathogen-specific IgG [35]. In HIV-infected individuals, gp140-specific memory B-cells are not only enriched in TLM B-cells but also in activated memory (AM, CD21^lo^CD27^+^) B-cells [36]. This latter population also accumulates in blood of chronically HIV-infected individuals [33] and is expanded in lymphoid organs of SIV-infected macaques [17,37]. In a cohort of individuals receiving seasonal influenza vaccine, AM B-cells were shown to be enriched in Ag-specific B-cells and harbor features of post-GC B-cells, prone to plasma cell differentiation but refractory to GC re-entry [38]. This latter observation is consistent with low expression of CD62L, CXCR4 and CXCR5 but increased expression of CXCR3 on CD21^lo^ memory B cells that preferentially guide them outside GC, as recently shown for T-bet^+^ B-cells in lymph nodes (LNs) of HIV-infected patients [39]. Based on these data, IFNγ overproduced by GC T_FH_ during HIV/SIV infection might orchestrate the CD21^lo^ B-cell trafficking by impairing chemokine receptor expression.

In an attempt to elucidate GC B-cell and T_FH_ dysfunctions in early HIV/SIV infection, here we propose a comprehensive study undertaken to compare GC reaction and humoral response in macaques either acutely infected by SIV or vaccinated with the Tetanus Toxoid (TT), a model TD vaccine Ag, known to elicit a lasting protective humoral response in humans and NHPs. The selection of TT as model Ag in this study relies on previous data in mice and humans, delineating the fate of TT-specific effector B-cells and Abs during primary and secondary immunization. After booster injection, TT-specific memory B-cells and plasma blasts/cells are widely distributed in human spleen, blood, tonsils and bone marrow [40] and helper T-cell response is polarized towards a Th1-cell phenotype as does the T-cell response to HIV-1 [41]. However, vaccination with TD Ags, including the TT vaccine or Env trimer-based vaccines, differs from HIV/SIV infection setting. First, SIV Ags, in particular Env, are dynamically modified over time during infection and displayed a broad set of epitopes whereas in the case of TT vaccine, TT Ag is only transiently present with a probably more stable set of epitopes. Additionally, a unique feature of HIV/SIV infection is CD4 T-cell depletion, particularly at the acute phase of infection. Nevertheless, it is worth underlining that young HIV-infected patients develop weaker antibody responses to vaccination with the trivalent seasonal influenza vaccine, despite virologic suppression and normal CD4 T-cell counts [42]. Apart, CD4 T-cell depletion, inflammation also lowers immune responses to Flu vaccine in HIV-infected and healthy individuals, with effector functions of blood B-cells and circulating T_FH_ being both impaired [43]. Therefore, a comparison of the impact of early viral infection and vaccination on GC reaction requires further investigations to understand whether GC T_FH_ expansion and functions are comparable to those occurring in response to vaccination to a conventional TD Ag. We paid a particular attention to changes in GC B- and T-cell actors and their organization, as well as in effector B-cells in spleen and LNs. Circulating titers of SIV-specific and TT-specific Abs and inflammatory cytokines were assessed simultaneously.

## 2. Materials and Methods

### 2.1. Animals, Infection and Immunization

Sixteen adult male cynomolgus macaques (*Macaca fascicularis*), each weighing about 4 kg and aging about 34 to 45 months, were imported from Mauritius (SILABE, Strasbourg, France) and housed in the accredited animal facilities of the Infectious Disease Models and Innovative Therapies (IDMIT) Infrastructure (Fontenay-aux-Roses, France). NHPs were handled in accordance to national regulations (Commisariat à l’Energie Atomique et aux Energies Alternatives (CEA) Permit Number A 92-32-02), in compliance with Standards for Human Care and Use of Laboratory of the Office for Laboratory Animal Welfare under Assurance number A5826-01, and the European Directive (2010/63, recommendation N°9). The study was approved by the Ministère de l’Education Nationale, de l’Enseignement Supérieur et de la Recherche (France) and the Comité d’Ethique en Expérimentation Animale n°44 under the reference 2015121509045664 (APAFIS#3178). Animals were fed standard monkey chow diet daily supplemented with fruit and vegetables and water ad libitum. Overall, animal health was daily monitored by care staff and veterinary personnel. The MHC haplotypes were determined (Table S1) and only animals negative for H6 haplotypes were used in this study. A first group of 10 macaques was intravenously inoculated with 5000 AID50 of a SIVmac251 stock [44] and followed for 28–30 days post-infection (pi) (Group SIV^+^). Experimental infection protocol and viral characterization of these SIV-infected macaques have been previously detailed in [17]. A second group of 6 macaques was immunized two times by intramuscular (IM) injection (0.5 mL per injection, pre-filled syringe) of a commercial Tetanus Toxoid (TT) vaccine (Sanofi Pasteur SA, Lyon, France). Bilateral immunizations in upper thighs were used to increase the magnitude of the TT-specific response. The first immunization was performed 40 days prior to the boost. Macaques were then sacrificed 21 days post-boost (dpb) (Group TT). All animals were sedated with ketamine hydrochloride (Merial SAS, Villeurbanne, France) before immunization, sample collection and necropsy.

### 2.2. Sample Collection and Processing

For serum/plasma sampling and complete blood count, blood was collected into serum clot activator tubes or into K3-EDTA tubes (both from Greiner Bio-One, Frickenhausen, Germany) from Group SIV^+^ before infection and at 2, 7, 10, 14, 21, 28/30 dpi; from Group TT before the prime and at 4, 7, 10, 14, 21, 28, 33 days post-priming, and before the boost (D0) and at 4, 7, 10, 14, 21 dpb. Aliquots of serum and plasma samples were kept frozen at −80°C until use. For both groups, blood cell formula and count, as well as hemoglobin concentration and hematocrit, were determined on K3-EDTA collected blood using an HMX A/L (Beckman Coulter, Villepinte, France). Spleen and inguinal LNs were immediately collected at necropsy under the supervision of veterinarians. Splenic biopsies were formalin-fixed and paraffin-embedded as previously described [45,46]. Splenic and nodular mononuclear cells were obtained by mechanical disruption in complete medium (RMPI 1640-glutamax medium supplemented with 1 mM sodium pyruvate, 100µg/mL streptomycin, 100 UI/mL penicillin, 10 mM HEPES, 2 mM non-essential amino acids and 10% heat-inactivated FCS, all from Invitrogen, Life Technologies SAS, Saint Aubin, France), filtered through a 70-µm-pore-size cell strainer and further purified by Ficoll gradient centrifugation. Cells were kept frozen in 90% FCS/10% DMSO in liquid nitrogen until use.

### 2.3. Quantification of Plasma Cytokines, Immunoglobulins and Antigen-Specific Antibodies

Serum CXCL13 was detected using Quantikine^®^ ELISA kits (Bio-Techne, Lille, France) with sensitivity limit of 3.97 pg/mL. Serum IFNα2 was detected using the Cynomolgus/Rhesus IFNα ELISA kit from PBL Assay Science (Bio-Techne) with a sensitivity limit of 25 pg/mL. Serum BAFF and CXCL10 were detected using a magnetic Luminex Kit (Bio-Techne) with sensitivity limits of 1.01 pg/mL and 1.18 pg/mL, respectively. Data were acquired using a Bioplex-200 and analyzed with the Bioplex Manager Software (Bio-Rad Laboratories, Redmond, WA, USA). Anti-SIV Abs (IgM plus IgG) were detected in plasma using Genscreen HIV1/2 ELISA kit, version 2 (Bio-Rad Laboratories, Redmond, WA, USA). Total IgM and IgG were quantified in serum by ELISA as previously described [45]. Homemade ELISA was used to quantify serum TT-specific IgM or IgG as previously reported [17]. For each macaque, plasma/serum samples, collected before and at different time points after infection or vaccination, were tested simultaneously and run in duplicates. Results are expressed as mean OD value (SIV- and TT-specific Abs) or as mean concentration (cytokines and total IgM/G).

### 2.4. Flow Cytometry Analyses

Panels of Abs used for multi-parameter FCM are shown in Tables S2 and S3. Optimized concentrations were predetermined for each Ab. For the detection of transcription factors (Bcl-6, Ki67), CD1c, CXCR3, surface receptors of BAFF (BAFF-R, TACI) and for intracellular detection of IFNγ, relevant Ab pairs with isotype controls were used and highlighted in tables in grey. After rapid thawing at 37°C and two washes in complete medium, cells were surface or intracellularly stained as previously described (17). For surface staining: 1 to 2 × 10^6^ cells in staining buffer (PBS 1X plus 0.5% BSA and 2 mM EDTA) were incubated with Live/Dead fixable blue stain (Invitrogen, Life Technologies SAS, Saint-Aubin, France) for 30 min at 4 °C before addition of 5% (vol/vol) heat-inactivated human AB serum for an extra 15 min at 4 °C. After washing, cells were labeled with appropriate Abs diluted in staining buffer for 30 min at 4 °C, then washed and fixed with 0.5% paraformaldehyde (PFA). For the detection of transcription factors, cells were first surface stained as above, and then fixed and permeabilized with the FoxP3/Transcription Factor staining buffer set (eBiosciences^TM^, Paris, France) before intracellular staining with appropriate monoclonal Abs for 45 min at 4 °C. Cells were washed twice and fixed in 0.5% PFA. For the detection of intracellular cytokines, cells (2 × 10^6^/mL) were stimulated for 5 hrs at 37 °C in 5% CO_2_ with PMA (50 ng/mL) and ionomycin (1 µg/mL) in the presence of Brefeldin A (BFA, 10 µg/mL) during the last 4 h before staining. After surface staining, cells were fixed with 2% PFA and treated with the BD cytofix/cytoperm kit before intracellular staining with anti-cytokine Abs. Events were acquired on a BD LSRII and data were analyzed using the Kaluza^®^ Flow Analysis Software (Beckman Coulter, Inc., Villepinte, France). Sphero^TM^ Rainbow calibration particles (BD Biosciences, Rungis, France) were used for daily calibration of the flow cytometer. Unstained cells and single-color beads were used for calculating the compensation matrix.

### 2.5. Immunohistochemistry and Digital Image Analysis

Sections (4μm-thick) were cut from formalin-fixed paraffin-embedded spleen blocks. Sections were subject to dewaxing, Ag retrieval, saturation and staining with various monoclonal and polyclonal Abs (Table S4) on a Leica-Bond III/Max autostainer platform (Leica Biosystems Nanterre, France). Detection of primary Ab binding was performed with bond compact polymer Refine detection (DAB, brown), containing substrate chromogen and hematoxylin counterstain.

Images of full section were generated on a Lamina Multilabel Slide Scanner (PerkinElmer, Gif s/Yvette, France), using the brightfield scan mode. Digital images were opened in Pannoramic Viewer software (v1.11.4, 3DHistech, Budapest, Hungary), and areas of interest were manually annotated using the drawing tools. For each section, all (or at least 10) random areas were extracted from the main scan for quantitative analysis. The software automatically calculated the sizes of the whole tissue and of the selected areas (in µm^2^). Quantification of positively labeled cells was performed with computer assisted image analysis using Inform (v2.3, 3DHistech, Budapest, Hungary) or Photoshop CS6 (Adobe Systems Inc., San Jose, CA) software by two independent investigators. Mean values of positive cells per area or per mm^2^ of tissue were calculated for each section and each macaque.

### 2.6. Statistical Analysis

All data were graphed and analyzed using Graphpad Prism (GraphPad Software, San Diego, CA, USA). Bar graphs represent Mean and SD. For pairwise comparisons, data were analyzed using Mann-Whitney U-test (unpaired, 2-tailed). One and 2-way ANOVA was used for multiple comparisons. Correlation coefficients were calculated using the Spearman rank test. Statistical significance is denoted on each figure.

## 3. Results

### 3.1. Humoral Response upon SIV-Infection or TT-Vaccination

**Figure 1:**
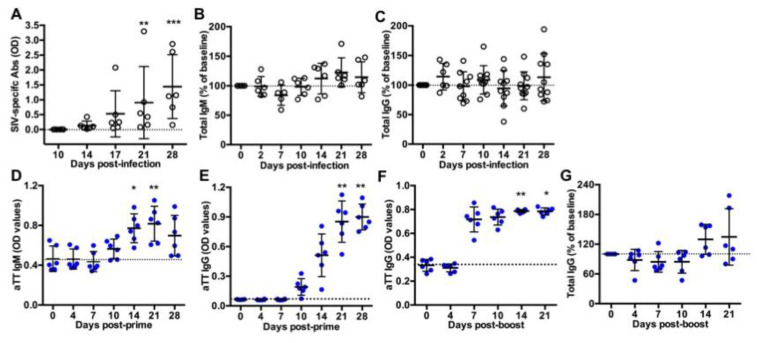
Titers of circulating antibodies in SIV-infected and TT-vaccinated macaques. ELISA was used to quantify (**A**) SIV-specific antibodies, (**B**) total IgM or (**C**) total IgG in plasma from SIV-infected macaques; (**D**) TT-specific IgM and (**E**) TT-specific IgG in serum from vaccinated macaques after the prime or (**F**) after the boost; and (**G**) total IgG in serum from TT-boosted animals. Values of specific TT- and SIV-specific Abs are given in OD values (**A**,**D**–**F**). Changes in total IgM and IgG concentration were expressed as the percentage of baseline value for each animal (**B**,**C**,**G**). A dotted line indicates the baseline value at D0. Each dot represents one macaque from Group SIV^+^ (*open circle*) or TT (*blue circle*). Bars represent Mean ± SD. Statistical comparison between values at D0 and at every time point was performed using Friedman test with Dunn’s multiple comparisons test. * p < 0.05, ** p < 0.01 and *** p < 0.001.

Ten macaques were intravenously inoculated with 5000AID50 SIVmac251 and followed for one month (acute phase of infection) before necropsy (Group SIV^+^). As previously reported, the kinetics of plasma viral load and CD4 T-cell counts in blood evidenced the viral infection and a comparable course of the disease in these macaques [17]. Titers of SIV-specific Abs progressively increased from day 14 post-infection (pi) but not yet reached a plateau value between days 21 and 28 pi (Figure 1A). At day 28 pi, we found no correlation between levels of SIV-specific Abs and either plasma viral loads or blood CD4 T-cell counts (data not shown). Compared to baseline values, total IgM (Figure 1B) and IgG (Figure 1C) titers did not significantly change during the acute phase of infection, suggesting a minor contribution of free SIV-specific Abs to total circulating Ig at that time.

Six macaques received a first IM injection of TT vaccine and a booster dose 40 days later (Group TT). This time point was thereafter considered as day 0 (D0) post-boost (pb). Titers of TT-specific IgM (Figure 1D) and IgG (Figure 1E) increased from day 10 post-prime. IgM titers peaked at day 21 post-prime with a slight non-significant decrease at day 28 (Figure 1D), whereas anti-TT IgG titers reached a plateau value from day 21 post-prime (Figure 1E). After the boost, levels of anti-TT IgG rapidly reached a plateau value from day 7 pb (Figure 1F), possibly due to a substantial differentiation of memory B-cells and PBs into plasma cells. Median value of total IgG titers increased by 33.5% at day 14 pb and by 13.5% at day 21 pb but these differences did not reach statistical significance (Figure 1G).

### 3.2. Systemic Inflammation upon SIV-Infection or TT-Vaccination

**Figure 2:**
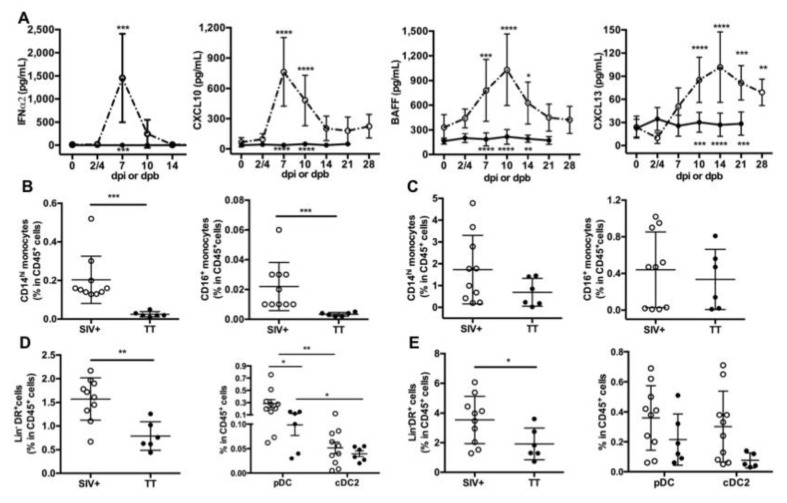
Circulating inflammatory cytokines and proportions of antigen-presenting cells in lymphoid organs. (**A**) ELISA was used to quantify serum levels of IFNα2 and CXCL13, whereas serum CXCL10 and BAFF were detected by using a magnetic Luminex kit. Samples were tested prior to infection or to booster injection (baseline value, D0) and at different time points post-infection (dpi) or post-boost (dpb). Mean ± SD values for group of SIV-infected macaques (*open circle*, *dotted line*) or TT-vaccinated macaques (*black circle; plain line*) at the various time points are given. Statistical comparison between values at D0 and at every time point post-infection (Group SIV^+^) or post-boost (Group TT) was carried using a Friedman test with Dunn’s multiple comparisons test. Statistically significant values are indicated above dotted lines. Comparison between values of each group at every time point was carried using 2-way ANOVA with Bonferroni’s multiple comparisons test. *p* values are indicated below the plain lines: ** p < 0.01, *** p < 0.001, and **** p < 0.0001. Proportions of CD14^hi^ and CD16^+^ monocytes in CD45^+^ cells from Group SIV^+^ and Group TT were compared in LNs (**B**) and spleen (**C**). Proportions of Lin^-^DR^+^ cells in CD45^+^ cells from Group SIV^+^ and Group TT are shown in LNs (**D**, left panel) and spleen (**E**, left panel). Proportions of pDC and cDC2 in CD45^+^ cells are given in LN (**D**, right panel) and spleen (**E**, right panel). For (**B**–**E**) each dot represents one macaque from Group SIV^+^ (*open circle*) or TT (*black circle*). Bars represent Mean ± SD. Statistical comparison between groups was performed using Mann Whitney non-parametric test. Statistically significant values are indicated * p < 0.05, ** p < 0.01 and *** p < *0.001*.

Inflammation at the site of virus infection or immunization is key for developing innate and adaptive immunity. HIV/SIV infection induces a storm of inflammatory cytokines during the acute phase with type I IFN contributing to the control of viremia, as shown in humanized mice [47]. In TT vaccine, Tetanus Toxoid is adsorbed on aluminum phosphate salts, forming a combination of Ag-adjuvant mandatory for initiating TT-specific Ab responses through local inflammation [48]. After IM vaccination of mice with TT, this local inflammation relies on cytokine/chemokine release, early recruitment of neutrophils followed by a more sustained recruitment of macrophages and dendritic cells (DCs). Recruitment of these cells is more pronounced after a booster injection [49]. Accordingly, we measured levels of blood inflammatory cytokines in both groups (Figure 2A). Consistent with our previous data [17], levels of IFNα2, CXCL10, BAFF and CXCL13 were elevated during the acute phase of SIV infection, with a peak at days 7 pi (IFNα2, CXCL10), 10pi (BAFF) or 14pi (CXCL13), respectively. In contrast to IFNα2 levels which returned to baseline value from day 14 pi, levels of CXCL10, BAFF and CXCL13 were still elevated at days 21 and 28 pi compared to baseline values. In TT-vaccinated macaques, serum IFNα2, CXCL10 and BAFF levels remained roughly unchanged over time after the prime (data not shown) and the boost (Figure 2A). After the booster dose, the highest CXCL13 value was observed between days 4 and 10 in most macaques (Figure S1) and the average value of the group increased by 53% at day 4 pb compared to pre-boost (34.6 pg/mL vs. 22.6 pg/mL) (Figure 2A and Figure S1). A modest increase was also observed at days 7 (22%) and 10 (20%) post-prime compared to pre-vaccination value (Figure S1). Collectively, this indicates that substantial titers of TT-specific IgG developed despite a limited inflammation at systemic level compared to SIV infection.

Since recruitment of APCs is influenced by inflammation at the site of immunization or infection, we sought to determine whether monocytes and DCs were differently represented in inguinal LNs and spleen from Groups SIV^+^ and TT at necropsy (day 28 pi and day 21 pb). In both lymphoid organs, CD14^hi^ monocytes outnumbered CD16^+^ monocytes. Frequencies of CD14^hi^ and CD16^+^ monocytes among CD45^+^ cells were significantly much higher in LNs from SIV-infected macaques than from TT-vaccinated macaques (Figure 2B). Whereas a trend to higher proportions of every monocyte subset was also observed in spleen from SIV-infected macaques, differences did not reach statistical significance (Figure 2C). Compared to TT-vaccinated macaques, total DCs (Lin^-^DR^+^ cells) were more frequent among CD45^+^ cells in LNs (Figure 2D, left panel) and spleen (Figure 2E, left panel) from SIV-infected macaques, suggesting a more potent recruitment of APCs in this group. Nodular pDC were more frequent than CD1c^+^DC (cDC2) in both settings, with significantly higher frequencies of pDC in group SIV^+^ than in group TT (Figure 2D, right panel). Consistent with monocytes releasing CXCL13 upon activation [50], serum CXCL13 titers correlated with the frequencies of CD14^hi^ and CD16^+^ monocytes in LNs but not in spleen (Table 1). Similarly, titers of IFNα, CXCL10 and BAFF correlated with frequencies of monocytes and total DCs in LNs, with pDC frequencies correlating with CXCL10 and BAFF titers only (Table 1). In spleen, such a correlation was found between CXCL10 titers and frequencies of CD14^hi^ and total DCs, and between BAFF titers and frequencies of total DCs and cDC2. Compared to TT vaccination, SIV infection is thus associated with a more intense APC mobilization and cytokine release. Despite lower inflammation, levels of TT-specific Abs raised rapidly from day 10 post-prime and from day 7 pb. Circulating SIV-specific Abs were only detectable from day 14 pi but their measurement during the two first weeks of infection is likely biased by their capture by high amounts of viral Ags resulting from increased virus production during this period. Consistently, SHIV-specific Abs are more rapidly detected in vaccination setting than after infection [13,14].

### 3.3. Differences in Nodular B-Cells and T_FH_ Associated with Responses to TT or SIV

**Figure 3:**
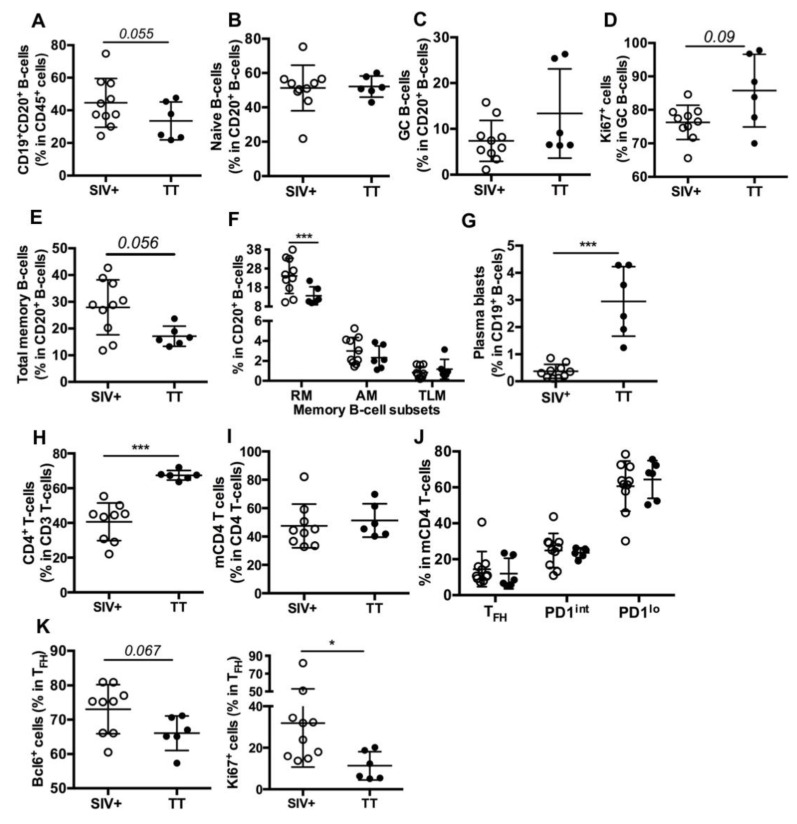
B- and T-cell subsets in inguinal LNs from SIV-infected and TT-vaccinated macaques. Cell proportions were determined by FCM for each macaque. Shown are proportions of (**A**) CD19^+^CD20^+^ B-cells (thereafter referred to as CD20^+^ B-cells) in nodular CD45^+^ cells; (**B**,**C**) naïve and GC B-cells in CD20^+^ B-cells; (**D**) Ki67^+^cells in GC B-cells; (**E**,**F**) Total memory, RM, AM and TLM B-cells in CD20^+^ B-cells; (**G**) plasma blasts in CD19^+^ B-cells; (**H**) CD4^+^ T-cells in CD3^+^ T-cells; (**I**) memory CD4 T-cells (mCD4 T-cells) in CD4^+^ T-cells; (**J**) T_FH_, PD1^int^ and PD1^lo^ subsets in mCD4 T-cells and **(K**) Bcl-6^+^ and Ki67^+^ cells in T_FH_ are shown. Each dot represents one macaque from the SIV^+^ (*open circle*) or TT (*black circle*) group. Bars represent Mean ± SD. For all panels except (**F**) and (**J**), statistical comparison between groups was performed using the Mann Whitney non-parametric test. For (**F**) and (**J**) panels, statistical comparison was performed using 2-way ANOVA with Bonferroni’s multiple comparisons test. Statistically significant values are indicated: * p < *0.05* and *** p < *0.001*.

We next compared the B-cell compartment in inguinal LNs from Groups SIV^+^ (day 28 pi) and TT (day 21 pb). Using polychromatic staining (Table S2, Panel 2) and the gating strategy depicted in Figure S2, we identified various B-cell subsets. Proportions of total B-cells (CD19^+^CD20^+^) in CD45^+^ cells were comparable in the Group TT (33.5% ± 4.7%) and in a group of healthy “control” macaques (36% ± 8.5%, *n* = 3) (data not shown). Compared to the Group TT, a trend to higher proportions of total B-cells (1.3-fold) was observed in the Group SIV^+^ (Figure 3A). When all macaques were considered, frequency of total B-cells inversely correlated with that of CD3^+^ T-cells (*r = −0.979*, *p* < *0.0001*) but not with that of CD4 T-cells (r *= −0.286*, *p = 0.301*) in CD45^+^ cells (data not shown).

Similar proportions of naïve B-cells (Figure 3B) were found in both groups but proportions of total GC B-cells (13.4% vs. 7.4%, Figure 3C) and of proliferating GC B-cells (86% vs. 76%, *p = 0.09*, Figure 3D) tended to be higher in the Group TT than in the Group SIV^+^. Lower proportions of total memory (Figure 3E) and RM B-cells (Figure 3F) were found in the Group TT whereas percentages of AM and TLM B-cells were similar in both groups (Figure 3F), with total CD21^lo^ memory B-cells representing 3.8 ± 0.52% and 3.5 ± 0.81% of B-cells in the Groups SIV^+^ and TT, respectively (data not shown). In contrast to RM B-cells, plasma blasts/cells (CD19^+^CD20^−^) were more frequent in TT-vaccinated macaques than in SIV-infected ones (Figure 3G). When all macaques were considered, percentages of GC B-cells correlated with those of plasma blasts/cells (*r = 0.55*, *p* < *0.03*) but not with those of total memory or RM B-cells (not shown). However, percentages of RM and plasma blasts/cells tended to inversely correlate (*r = −0.494*, *p = 0.054*). We thus infer that higher PB proportions in the LNs of boosted macaques rely on enhanced extrafollicular differentiation of RM B-cells into PBs and on biased generation of PBs over RM B-cells in secondary GC.

Consistent with SIV infection depleting CD4^+^ T-cells, their proportions were lower in SIV-infected macaques than in TT-vaccinated ones (Figure 3H). Based on the expression levels of PD1 and ICOS (Table S2, Panel 3) on mCD4 T-cells, we distinguished three subsets referred to as T_FH_, PD1^int^ and PD1^lo^ (Figure S3). Despite similar proportions of mCD4 T-cells (Figure 3I), nodular mCD4 T-cells preferentially exhibited a PD1^lo^ phenotype in both groups, with only 14.5% and 12% being T_FH_ in the Groups SIV^+^ and TT, respectively. No significant difference between groups was observed for each mCD4 T-cell subset (Figure 3J). Frequencies of GC B-cells did not correlate with those of T_FH_ in mCD4 T-cells but correlated with frequencies of T_FH_ in CD4 T-cells (*r = 0.609*, *p* < *0.02*) (not shown). Higher frequencies of Bcl-6^+^ and Ki67^+^ cells were found in T_FH_ from the Group SIV^+^ compared to the Group TT (Figure 3K), possibly indicating less active T_FH_ in this later group. With less APCs being present in LNs from boosted macaques and lower inflammation (Figure 2D), polarization of CD4 T-cells into T_FH_ is likely less strong and enduring in vaccinated macaques than in SIV-infected ones. Indeed, T_FH_ themselves constitute a persistent source of viral Ags in GC of SIV-infected macaques, whereas TT concentrations progressively wane.

### 3.4. Differences in Splenic B-Cells Associated with Responses to TT or SIV

**Figure 4:**
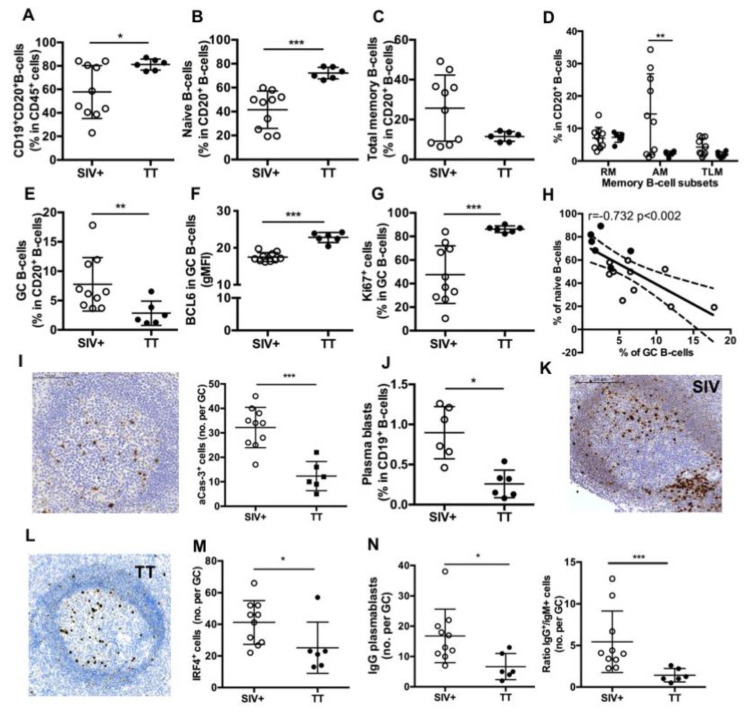
Splenic B-cell subsets in SIV-infected and TT-vaccinated macaques. (**A**) Proportions of CD19^+^CD20^+^ B-cells in CD45^+^ cells as well as proportions of (**B**) naïve, (**C**) Total memory B-cells; (**D**) RM, AM and TLM B-cells; and (**E**) GC B-cells in CD20^+^ B-cells were determined by multi-parameter FCM for each macaque. (**F**) Intensity of Bcl-6 expression (gMFI) and (**G**) proportions of Ki67^+^ cells in GC B-cells are shown. (**H**) Graph represents the correlation between the percentages of GC and naïve B-cells. Spearman rank test was used for statistical analyses. *rho* and *p* values are given. (**I**) Representative staining with active-caspase-3 (aCas-3) Ab on section of one macaque from Group TT. Scale Bar = 200 µm (left panel), graph represents the Mean number of aCas-3^+^ cells per GC for each macaque (right panel). (**J**) Proportions of plasma blasts (PBs) in CD19^+^ B-cells are shown. (**K**,**L**) Representative IRF4 staining on sections of one representative macaque from Groups (**K**) SIV^+^ and (**L**) TT. Scale Bar = 200 µm. (**M**) Graph represents the Mean number of IRF4^+^ cells per GC for each macaque. (**N**) Graph represents the Mean number of IgG^+^ cells per GC for each macaque (left panel). Ratio between numbers of IgG and IgM PBs per GC was plotted for each macaque (right panel). In all panels, each dot represents one macaque from Groups SIV^+^ (*open circle*) or TT (*black circle*). For all panels except (**H**), bars represent Mean ± SD. For all panels except (**D**) and (**H**), statistical comparison between groups was performed using Mann Whitney non-parametric test. For panel (**D**), statistical comparison between groups was performed using 2-way ANOVA with Bonferroni’s multiple comparisons test. Significant statistical values are indicated: * p < *0.05*, ** p < *0.01* and *** p < *0.001*.

As evidenced on spleen section stained with CD20 Ab, surface of B-cell areas was comparable between the Groups SIV^+^ and TT (19.2 ± 1.6% and 17.4 ± 2.5% of the total tissue, respectively) (not shown). However, CD19^+^CD20^+^ B-cells were 38% more frequent among CD45^+^ cells in the Group TT than in the Group SIV^+^ (Figure 4A), with a 1.75-fold more naïve B-cells (Figure 4B) and obvious lower proportions of total (Figure 4C) and AM memory B-cells (Figure 4D).

By using IHC approach and Ki67 staining, we found that frequencies of active follicles (follicles with GC) were not different between both groups (54.9 ± 4.8% in the Group SIV^+^ and 46.3 ± 4.6% in the Group TT), but the area occupied by GC within follicle was 30% lower in the Group TT than in the Group SIV^+^ (not shown). FCM analysis showed that proportions of GC B-cells in total B-cells were 63% lower in the Group TT than in the Group SIV^+^ (Figure 4E) but exhibited higher expression of Bcl-6 (gMFI) (Figure 4F) and contained a higher percentage of proliferating (Ki67^+^ cells) B-cells (Figure 4G). No difference in the intensity of Ki67 expression (average gMFI was 10.7 ± 1.1 for the Group SIV^+^ vs. 13.5 ± 1.4 for the Group TT) was concurrently observed (data not shown). When all macaques were considered, frequency of GC B-cells inversely correlated with that of naïve B cells (*r = −0.732*, *p* < *0.002*) (Figure 4H). A three-fold increase in the number of apoptotic cells (aCas-3^+^ cells) was detected in GC from the Group SIV^+^ compared to the Group TT (Figure 4I), with most of them being CD3-negative cells (data not shown). Thus, one third of GC B-cells failed to develop into effectors in the Group SIV^+^. Numbers of aCas-3^+^ cells positively correlated with frequencies of total GC B-cells (*r = 0.634*, *p* < *0.01*) but inversely with those of proliferating GC B-cells (*r = −0.622*, *p* < *0.02*) (not shown).

Plasma blasts/cells were significantly less frequent in the Group TT than in the Group SIV^+^ (Figure 4J). When PBs were quantified in GC by using IHC and IRF4 staining, we consistently found less total PBs per GC in TT-vaccinated macaques than in SIV-infected ones (Figure 4K–M). This difference essentially relied on lower numbers of IgG PBs in the TT group (Figure 4N). Accordingly, the average value of IgG to IgM ratio was 5.4 and 1.4 in the Group SIV^+^ and Group TT, respectively. Compared to response to SIV Ags, response to TT is thus associated with less PBs and AM B-cells in spleen. Frequency of GC B-cells correlated with that of PBs (*r = 0.634*, *p* < *0.03*) (not shown). In contrast to TT antigen transiently exposing a define pool of B-cell epitopes, SIV likely exposes a more diversified pool of antigens that persists and dynamically evolves during the first month of infection. This might contribute to a broader stimulation of B-cells entering GC and generating PBs.

### 3.5. Major Differences in Splenic T_FH_ and Other mCD4 T-Cell Subsets between the Two Groups

**Figure 5:**
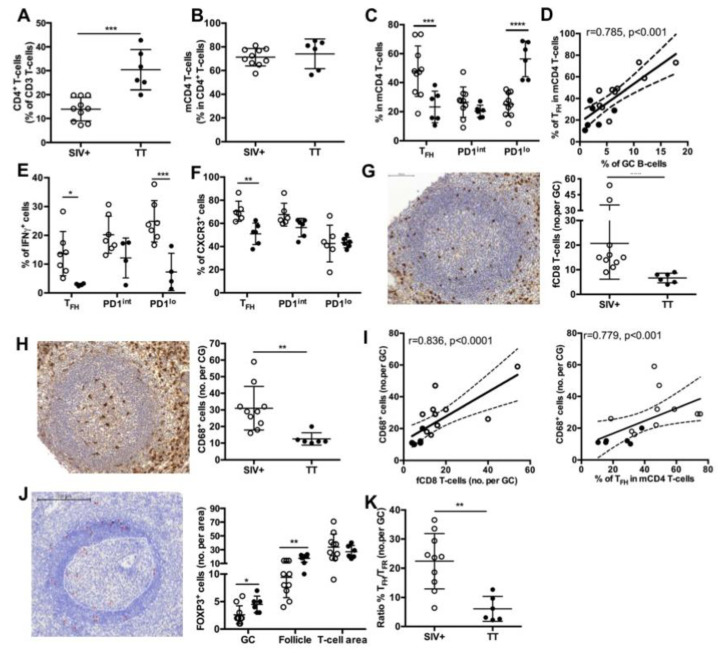
Splenic T-cell subsets in SIV-infected and TT- vaccinated macaques. Proportions of (**A**) CD4^+^ T-cells in total T-cells and (**B**) mCD4 T-cells in CD4^+^ T-cells are shown. (**C**) Proportions of T_FH_, PD1^int^ and PD1^lo^ subsets for each macaque are shown. Statistical comparison between groups was performed using 2-way ANOVA with Bonferroni’s multiple comparisons test. Statistically significant values are indicated: *** *p* < 0.001, **** p < 0.0001. (**D**) Correlation between the percentages of GC B-cells and T_FH_ is shown. Spearman rank test was used for statistical analyses. *rho* and *p* values are given. (**E**) Proportions of IFNγ^+^ cells in every mCD4 T-cells are plotted for each macaque. Seven and four macaques have been tested for the Group SIV^+^ and TT, respectively. Statistical comparison between groups was performed using 2-way ANOVA with Bonferroni’s multiple comparisons test. Statistically significant values are indicated: * p < 0.05 and *** p < *0.001.* (**F**) Proportions of CXCR3^+^ cells in every mCD4 T-cell subset are plotted for each macaque. Six macaques have been tested for each group. Statistical comparison between groups was performed using 2-way ANOVA with Bonferroni’s multiple comparisons test. Statistically significant values are indicated: ** p < *0.01.* Representative staining with (**G**) CD8 and (**H**) CD68 Abs of spleen sections from one macaque of the TT group is shown in left panels. Mean numbers of positive cells per GC are plotted for each macaque (right panels). Scale Bar = 100 µm (**I**) Graph represents the correlation between the number of CD68^+^cells per GC and either the number of fCD8 per GC (left panel) or the percentages of T_FH_ (right panel). Spearman rank test was used for statistical analyses. *rho* and *p* values are given. Each dot represents mean value for one macaque from the SIV^+^ (*open circle*) or TT (*black circle*) group. (**J**) Representative staining with FOXP3 Ab of spleen sections from one macaque of the TT group is shown in left panel. GC and mantle zone are fixed by white dotted lines and FOXP3^+^ cells are indicated by red asterisks. Proportions of FOXP3^+^ cells in GC, follicle and T-cell area are plotted for each macaque. Scale Bar = 200 µm. (**K**) Ratio between numbers of T_FH_ and T_FR_ per GC was plotted for each macaque from Groups SIV^+^ and TT. Statistical comparison between groups was performed using 2-way ANOVA with Bonferroni’s multiple comparisons test. Statistically significant values are indicated: * p < *0.05* and ** p < *0.01.* For (**A**,**B**,**G**,**H**,**K**), bars represent Mean values of the group ± SD and statistical comparison between groups was performed using the Mann Whitney non-parametric test. Statistically significant values are indicated: ** p < *0.01* and *** p < *0.001*.

As previously observed in LNs (Figure 3H), percentages of CD4^+^ T-cells among T-cells were reduced in spleen from the Group SIV^+^ compared to the Group TT (Figure 5A), with similar proportions of mCD4 T-cells (Figure 5B). Among mCD4 T-cells, SIV infection and TT vaccination were associated with a different balance between T_FH_ and PD1^lo^ cells (Figure 5C). Indeed, proportions of T_FH_ were significantly lower in the Group TT compared to the Group SIV^+^, whereas an opposite trend was observed for the PD1^lo^ subset. However, comparable percentages of Bcl-6^+^ and Ki67^+^ cells were found among T_FH_ in both settings (data not shown). When all macaques were considered, proportions of splenic T_FH_ significantly correlated with those of GC B-cells (Figure 5D) and with levels of inflammatory cytokines, in particular CXCL13 which can guide T_FH_ recruitment into GC (Table 2).

To further evaluate potential difference in T_FH_ polarization between the two groups, we first examined the intracellular expression of IFNγ in various mCD4 T-cell subsets (Table S2, panel 4). IFNγ-expressing cells represented 14% of T_FH_ in SIV-infected macaques but only 3% in TT-vaccinated macaques. However, higher proportions of IFNγ^+^ cells were present in PD1^lo^ and PD1^int^ mCD4 T-cells than in T_FH_ in both settings. Indeed, 25% of PD1^lo^ and 20% of PD1^int^ mCD4 T-cells were IFNγ^+^ in SIV-infected macaques, whereas only 7.3% and 12% of these subsets were IFNγ^+^ in the Group TT (Figure 5E). Alternatively, we examined the expression of CXCR3 in T_FH_ and other mCD4 T-cells (Table S2, panel 5). Consistent with data on IFNγ expression in T_FH_, CXCR3^+^ T_FH_ (T_FH1_-like) were significantly more frequent in the Group SIV^+^ than in the Group TT (70.5 ± 3.6% vs. 51 ± 3.5%, respectively) (Figure 5F).

We have recently shown that follicular CD8^+^ (fCD8) T-cells infiltrate GC as soon as the acute phase of SIV infection [17]. As expected, TT vaccination was characterized by lower numbers of fCD8 per GC compared to SIV infection (Figure 5G). Similar results were observed when positive cells per mm^2^ of GC area were considered (not shown). Consistent with a role of monocytes-mediated inflammation in the recruitment of fCD8 T-cells, number of infiltrating macrophages (CD68^+^ cells) in GC, that was significantly higher in the Group SIV^+^ than in the Group TT (Figure 5H), correlated with numbers of fCD8 but also with frequency of T_FH_ (Figure 5I). In addition, numbers of fCD8 in GC correlated with CXCL10 and CXCL13 titers (Table 2). Follicular regulatory T-cells (T_FR_, FOXP3^+^) were rare in GC in both settings but higher number per GC and B-cell follicle were found in the Group TT (Figure 5J). Accordingly, the ratio between T_FH_ and T_FR_ was significantly lower in TT-boosted animals compared to the Group SIV^+^ (6.1 ± 1.8% vs. 22.4 ± 3%) (Figure 5K).

### 3.6. BAFF-R and TACI Expression on GC B-Cells and T_FH_

**Figure 6:**
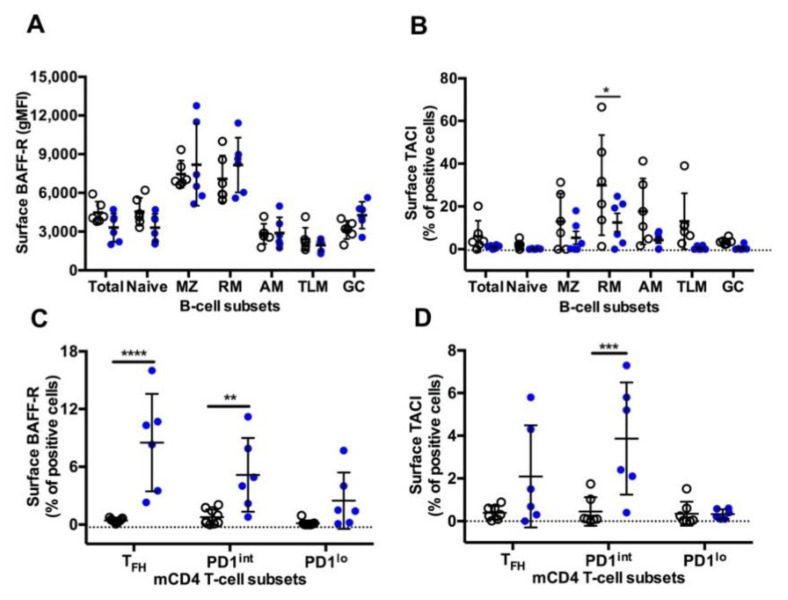
BAFF-R and TACI expression on splenic B-cells and mCD4 T-cell subsets from Groups SIV^+^ and TT. Surface expression of (**A**) BAFF-R (gMFI) (Table S3, Panel 5) and (**B**) TACI (% of positive cells) (Table S3, Panel 6) was analysed in every B-cell subset from macaques of SIV^+^ (*n* = 6*)* and TT (*n* = 6*)* groups. Surface expression of (**C**) BAFF-R (Table S3, Panel 7) and (**D**) TACI (Table S3, Panel 8) (% of positive cells) was simultaneously determined in every mCD4 T-cell subset from macaques of SIV^+^ (*n* = 9*)* and TT (*n* = 6*)* groups. Each dot represents one macaque from the SIV^+^ (*open circle*) or TT (*blue circle*) group. Bars represent Mean ±SD. Statistical comparison between groups was performed using 2-way ANOVA with Bonferroni’s multiple comparisons test. Statistically significant values are indicated: * *p < 0.05*, ** p < *0.01*, *** p < 0.001 and **** p < *0.0001.*

A similar pattern of surface BAFF-R expression was observed on B-cells in both settings, with higher expression on MZ and RM B-cells compared to other B-cell subsets (Figure 6A). As we previously reported [17], the highest frequency of TACI^+^ cells was found in RM B-cells, with intermediate frequencies on MZ, AM and TLM B-cells and less than 5% positive cells in naïve and GC B-cells (Figure 6B). Compared to the Group SIV^+^, expression of TACI was lower in all B-cell subsets from the Group TT, but a significant difference was only reached in RM B-cells. While in SIV-infected macaques BAFF-R and TACI were expressed by less than 1% of cells in the various subsets of mCD4 T-cells, all mCD4 T-cell subsets expressed higher level of BAFF-R in TT-vaccinated animals, with an average of 8.5% of BAFF-R^+^ cells among T_FH_ cells (Figure 6C). In the group TT, TACI was present on 2.1% and 3.9% of T_FH_ and PD1^int^ T-cells, respectively (Figure 6D). This suggests that T_FH_ and PD1^int^ are responsive to BAFF during TT vaccination but lost this ability during SIV infection.

## 4. Discussion

Our present data indicate that the extend of blood inflammation strongly differs between the two settings, mainly pro-inflammatory in SIV-infected macaques as previously reported [17,51,52] but more restricted after primary and secondary immunization with TT. CXCL13 is considered as a reliable marker of GC activity [53] but its systemic levels remain globally low in our vaccinated macaques despite functional GC and substantial production of TT-specific IgG. Although modest, the rise in CXCL13 levels that occurred by days 7–10 post-prime in most macaques (5–6 pg/mL, 40% over the pre-vaccination value, Figure S1) and by day 4 pb (32 pg/mL, 60% over pre-boost value) might be physiologically significant. The kinetics and the magnitude of increase were consistent with those observed in healthy individuals vaccinated against Yellow Fever or with Adenovirus 5-vector encoding HIV-1 Env proteins, as well as in macaques after the second or third immunization with adjuvanted SIVmac Env protein [13]. In our hands, serum CXCL13 titers correlated with the frequencies of nodular CD14^hi^ and CD16^+^ monocytes and spleen T_FH_, suggesting that both T_FH_ and monocytes could contribute to elevated CXCL13 blood levels. In SIV-infected macaques, we have previously shown that sorted T_FH_ and PD1^int^ produced CXCL13 and IL21 [17]. In line with T_FH_ enhancing sequestration of GC B-cells through CXCL13 release, a positive correlation was observed between CXCL13 titers and frequencies of spleen GC B-cells (*r = 0.4735*, *p* < *0.01*). Conflicting results have been reported regarding CXCL13 as a predictive marker of bNAbs in various groups of HIV-infected individuals [53,54,55,56,57]. Although we did not assess the neutralizing activity of Abs in this study we found no correlation between levels of systemic CXCL13 and those of SIV-specific Abs or TT-specific IgG (data not shown).

It is now well established that neutrophils, myeloid cells and DC are potent sources of CXCL10 during various settings. CXCL10 titers were strongly elevated in SIV-infected macaques but not in TT-vaccinated macaques, with titers correlating with frequencies of CD14^hi^ monocytes and total DC in LNs and spleen but also with frequencies of nodular pDC and numbers of macrophages (CD68^+^) in spleen GC. Heightened levels of SIV-induced IFNα likely enhance CXCL10 and CXCL13 production by Ag-presenting cells. As previously suggested [58,59], CXCL10 produced by infiltrating monocytes and/or DC could synergize with CXCL13 to attract/sequester T_FH_—about 70% of them expressing CXCR3 in our SIV-infected macaques—but also fCD8 in GC of SIV-infected macaques. Accordingly, numbers of fCD8 correlated with those of infiltrating macrophages, both numbers being significantly lower in the Group TT than in the Group SIV^+^. Numbers of fCD8 correlated with those of apoptotic cells in GC (*r = 0.737*, *p* < *0.02*, not shown) suggesting that fCD8 might exert cytolytic functions. T_FR_ are more numerous in spleen GC of TT-vaccinated macaques with a T_FH_/T_FR_ ratio increased by 3.7-fold compared to the Group SIV^+^. A higher ratio might strengthen the selection of TT-specific B-cells with higher BCR affinity [20]. In agreement with data of Sayin et al. [60], we found that T_FR_ preferentially reside at the border between B-cell follicle and T-cell area or between GC and mantle zone in both settings suggesting T_FR_ can exert regulatory functions in GC but also on early activation of GC B-cell founders and pre-T_FH_ at the outside border of follicle.

In agreement with data in healthy individuals revaccinated by TT [40,61], serum TT-specific IgG reached a plateau value at day 7 pb in our TT-vaccinated macaques which is consistent with a rapid extrafollicular maturation of plasma blasts and/or memory B-cells into plasma cells [62]. With more than 80% proliferating cells in spleen and nodular GC B-cells from TT-vaccinated macaques, GC reaction was likely restarted by the boost, as indicated by the presence of PBs in spleen GC and high frequencies of plasma blasts/cells in lymph node at day 21 pb. A majority of spleen and LN memory B-cells exhibited a RM phenotype in TT-vaccinated macaques, while half of memory B-cells had a phenotype of AM B-cells in spleen from SIV-infected macaques. In agreement with data showing that T-bet^+^ CD21^lo^ memory B-cells preferentially accumulate in extrafollicular areas of LN in chronically HIV-infected patients [39], most T-bet^+^ cells (B- and T-cells) were localized outside GC in spleen from SIV-infected and TT-vaccinated macaques. Only rare T-bet^+^ cells were found in GC, even in SIV-infected macaques (data not shown). Splenic T_FH_ in SIV-infected macaques simultaneously producing IFNγ (this work and [17]) and viral Ags could license the generation of AM B-cells in GC, as suggested by others [30,39]. Moreover, spleen AM B-cells are less frequent in TT-vaccinated macaques, with less T_FH_ expressing IFNγ. Supporting a link between IFNγ production in GC and generation of AM B-cells, the frequencies of IFNγ^+^ in T_FH_ correlated with those of AM B-cells in total B-cells (*r = 0.90*, *p* < *0.0001*, not shown). In SIV-infected macaques, elevated proportions of AM and TLM B-cells unable to re-enter GC might lower affinity maturation of SIV-specific Abs. Contrasting with substantial accumulation of AM in spleen from SIV-infected macaques compared to TT-vaccinated macaques, proportions of AM were comparable in LNs for both settings. At least during acute infection, nodular AM B-cells could promptly exit into blood and transiently accumulate in spleen areas in close contact to blood (marginal zone or red pulp) due to different trafficking constraints.

The pattern of BAFF-R expression is comparable on B-cell subsets between the two settings, with a lower expression on GC and atypical memory B-cells. This latter result extends pioneer observations showing that BAFF-R expression is decreased on CD21^lo^ blood B-cells in viremic HIV-infected patients, impairing their in vitro survival in response to BAFF [63]. However, the CD21^lo^ population examined in this later paper was heterogenous including immature and mature B-cells and possibly plasma blasts/cells. In contrast to B-cells, mCD4 T-cells differently express BAFF-R in the two settings. Indeed, BAFF-R is absent from mCD4 T-cell subsets in SIV-infected macaques (this work and [17]), whereas it is expressed on T_FH_ and, to a lesser extent, on PD1^int^ and PD1^lo^ mCD4 T-cells in TT-vaccinated animals. A low percentage of TACI^+^ cells is also found in these three subsets, suggesting that the low BAFF levels are insufficient at inducing the cleavage or internalization of BAFF receptor [64]. Expression of BAFF-R on circulating T_FH_ has been previously reported in patients with lupus but was associated with increased BAFF levels [65]. Whereas BAFF/BAFF-R interactions induce IFNγ release by T_FH_ in lupus-prone mice, the production of IFNγ by GC T_FH_ was not modified by in vivo BAFF neutralization in SIV-infected macaques [17]. Thus BAFF-R is differently expressed by T_FH_ during response to a TD Ag or viral infections, and BAFF/BAFF-R interactions on T_FH_ differently regulate IFNγ in autoimmune and infection settings. In vaccine settings, BAFF produced at low levels in GC might nevertheless contribute to T_FH_ physiology and GC reaction.

Thus, a compromised GC reaction and a potent inflammation characterize SIV infection compared to TT vaccination. In spleen and even in inguinal LNs, near the site of immunization, monocytes and DCs are less frequent in TT-vaccinated macaques than in SIV-infected ones, which probably results from Ag persistence and potent inflammation in the latter group. Spleen GC reaction is of higher magnitude in SIV-infected macaques but several impairments could limit the induction of a protective immunity. First, virus-induced CD4 T-cell loss might reduce the renewal of GC T_FH_ and their ability to establish appropriate cognate interactions with GC B-cells at the border of follicles or thereafter in GC. Second, helper functions of T_FH_ could be impaired by their polarization into T_FH1_-like, by inflammation-induced recruitment of fCD8 (either regulatory or cytotoxic) and by low numbers of T_FR_ limiting T_FH_/T_FR_ competition for high affinity B-cells. Third, the IFNγ-enriched GC microenvironment likely favors accumulation of spleen AM B-cells that no more participate to the GC reaction and the Ab affinity maturation. Increased GC B-cells apoptosis might also contribute to eliminate SIV-specific B-cells. With GC B-cells expressing low levels of BAFF-R in both settings, it seems unlikely that BAFF contributes to their survival or selection. In contrast, BAFF-R and TACI being expressed by T_FH_ in vaccinated macaques, BAFF might interfere with T_FH_ physiology.

## Figures and Tables

**Table 1:** Monocytes and DC: Correlation with inflammation.

		Lymph Node		Spleen	
		Spearman Rank Test ^a^		Spearman Rank Test ^a^	
**Cytokine ^b^**	Cell Subset ^c^	*p Values*	*rho*	*p Values*	*rho*
	CD14^hi^ monocytes	*	0.5801	*ns*	
	CD16^+^ monocytes	**	0.640	*ns*	
**CXCL13**	Total DC ^d^	*ns*		*ns*	
	cDC2	*ns*		*0.064*	0.492
	pDC	*ns*		*ns*	
	CD14^hi^ monocytes	*	0.611	*ns*	
	CD16^+^ monocytes	*	0.571	*ns*	
**IFN** **α**	Total DC	*	0.567	*ns*	
	cDC2	*ns*		*ns*	
	pDC	*ns*		*ns*	
	CD14^hi^ monocytes	**	0.737	*	0.555
	CD16^+^ monocytes	***	0.767	*ns*	
**CXCL10**	Total DC	*	0.603	*	0.632
	cDC2	*ns*		*ns*	
	pDC	*	0.616	*ns*	
	CD14^hi^ monocytes	**	0.748	*0.056*	0.491
	CD16^+^ monocytes	***	0.811	ns	
**BAFF**	Total DC	*	0.539	*	0.569
	cDC2	ns		**	
	pDC	**	0.677	ns	0.690

^a^ Spearman rank test was used for testing correlation. Sixteen animals were considered for all analyses. Statistically significant values are indicated: * *p* < *0.05*, ** p < *0.01* and *** p < *0.001*; *ns*, not significant; ^b^ For CXCL13, titers at day 4 pb for the TT group and at day 14 pi for the SIV^+^ group were used. Titers at day 7 (IFNα) and day 10 (CXCL10, BAFF) pi or pb were used. ^c^ Cell subsets as percent of CD45^+^ cells. ^d^ Total DC subset corresponds to Lin^-^DR^+^ cells.

**Table 2:** Correlation between cytokine titers in blood and frequencies of T-cell subsets and monocytes in spleen GC.

Subset	Cytokine ^a^	Spearman Rank Test ^b^	
		*p Values*	*Rho*
	IFNα	**	0.761
T_FH_	CXCL10	**	0.743
% in mCD4 T-cells	CXCL13	*	0.571
	IFNα	****	0.841
CD68^+^ cells	CXCL10	****	0.853
no. per GC	CXCL13	*ns*	
	BAFF	**	0.734
	IFNα	****	0.854
fCD8 T-cells	CXCL10	**	0.730
no. per GC	CXCL13	*	0.584

^a^ Titers were considered at day 7 pi or pb for IFNα and at day 10 pi or pb for BAFF and CXCL10. CXCL13 titers were considered at day 4 pb and day 14 pi for the groups TT and SIV^+^, respectively; ^b^ Ten SIV-infected macaques and 6 TT-vaccinated macaques were considered for correlation analysis. Statistically significant values are indicated: * *p < 0.05*, ** p < 0.01 and **** p < 0.0001; *ns*, not significant.

## Data Availability

Not Applicable.

## Supplementary Materials

The following are available online at https://www.mdpi.com/1999-4915/13/2/263/s1. Figure S1: Levels of Serum CXCL13 during TT-Immunization. Figure S2: Gating Strategy for the Identification of B-Cell Subsets in Lymphoid Organs. Figure S3: Gating Strategies for the Identification of Memory CD4 T-Cell Subsets. Table S1: MHC haplotypes of enrolled macaques. Table S2: Antibody Panels for Multiparameter Flow Cytometry. Table S3: Detection of BAFF Receptors on B- and T-cells by Flow Cytometry. Table S4: Antibodies for Immunohistochemistry.
