# Supplementary material for: Distinct Features of Germinal Center Reactions in Macaques Infected by SIV or Vaccinated with a T-Dependent Model Antigen
Source: Viruses. 2021 Feb 9;13(2):263. doi: 10.3390/v13020263 (PMC7916050; doi:10.3390/v13020263)
Supplement: Supplementary file 1 [file viruses-13-00263-s001.zip › viruses-1062458. suppl zip/Legends to Supplematary Figures.pdf]

Legends to supplemental Figures

Figure S1. Levels of serum CXCL13 during TT-immunization.

ELISA was used to quantify levels of serum CXCL13 in TT-vaccinated macaques. Samples were collected before and after the prime injection (*black triangle, dotted line*) and before and after the booster dose (*black circle, plain line*). Mean value ±SEM of the group at each time point is given. Statistical comparison between values before and after (prime or boost) vaccination was carried using a Friedman test with Dunn's multiple comparisons test but values did not reach statistical significance.

Figure S2. Gating strategy for the identification of B-cell subsets in lymphoid organs.

Panel 2 (**Table S1**) was used to analyse the composition of the B-cell pool present in spleen and lymph node. Given are representative dot plots showing the gating strategy used to identify various B-cell subsets in live $CD45^+$ B-cells: within $CD20^+Bcl\text{-}6^+$ B-cells depicted are GC B-cells; within $CD20^+Bcl\text{-}6^-$ B-cells depicted are naïve B cells ($SIgD^+CD21^+$), marginal zone (MZ) B-cells ($SIgD^+CD21^{hi}$), resting memory B-cells (RM, $SIgD^-CD21^+$), activated memory (AM, $SIgD^-CD21^-CD27^+$) and tissue-like memory (TLM, $SIgD^-CD21^-CD27^-$) B-cells.

Figure S3. Gating strategies for the identification of memory CD4 T-cell subsets.

Given are representative dot plots showing the gating strategy used to identify memory CD4 T-cell subsets in live $CD45^+$ T-cells. The gating strategy is shown for one SIV-infected macaque. Spleen cells were either stained with (**A**) Panel 3 (PD1, ICOS) or (**B**) Panel 5 (PD1, CXCR5) of **Table S1**. Accordingly, T_{FH} subset corresponds to either $PD1^{hi}ICOS^{hi}$ or $PD1^{hi}CXCR5^{hi}$; $PD1^{int}$ subset corresponds to either $PD1^{int}ICOS^{int}$ or $PD1^{int}CXCR5^{int}$ and $PD1^{lo}$ subset corresponds to either $PD1^{lo}ICOS^{lo}$ or $PD1^{lo}CXCR5^{lo}$.
