# Supplementary material for: Distinct Features of Germinal Center Reactions in Macaques Infected by SIV or Vaccinated with a T-Dependent Model Antigen
Source: Viruses. 2021 Feb 9;13(2):263. doi: 10.3390/v13020263 (PMC7916050; doi:10.3390/v13020263)
Supplement: Supplementary file 1 [file viruses-13-00263-s001.zip › viruses-1062458. suppl zip/Table S1. MHC.pdf]

Table S1. MHC haplotypes of macaques

SIV^{+} (n=10)	TT (n=6)
H2/H1	H2/H5
H2/RecH1H4	H1/RecH2H3H1
H2/H4	H2/H4
RecH3H2/RecH2H1	H1/H1
H3/RecH1H5	H1/RecH3H5
H3/H5	H3/RecH3H4
H3/H2	
H3/H1 (2 macaques)	
H3/RecH3H1	
