# Supplementary material for: Distinct Features of Germinal Center Reactions in Macaques Infected by SIV or Vaccinated with a T-Dependent Model Antigen
Source: Viruses. 2021 Feb 9;13(2):263. doi: 10.3390/v13020263 (PMC7916050; doi:10.3390/v13020263)
Supplement: Supplementary file 1 [file viruses-13-00263-s001.zip › viruses-1062458. suppl zip/Table S2-FCM1.pdf]

Table S2. Antibody panels for multiparameter flow cytometry

Panel 1 – Monocyte & DC subtypes

Fluorescent dye	*Antigen*	*clone*	*Manufact* [a]
Blue stain (UV)	**Live-Dead**	/	Invitrogen
PerCP	**CD45**	D058-1283	BD
V500	**CD3**	SP34.2	BD
AlexaFluor700	**CD20**	2H7	BLE
Pe-Cy7	**CD14**	M5E2	BD
APC-H7	**CD16**	3G8	BD
ECD	**HLA-DR**	Immu357	Coulter
FITC	**BDCA-2**	AC144	MACS
PE	**CD123**	7G3	BD
APC	**CD1c**	AD5-8E7	MACS
APC	**mIgG2a**	S43.10	MACS

Panel 2 – B-cell subsets in lymphoid organs

Fluorescent dye	*Antigen*	*clone*	*Manufact* [a]
Blue stain (UV)	**Live-Dead**	/	Invitrogen
PerCP	**CD45**	D058-1283	BD
V500	**CD3**	SP34.2	BD
AlexaFluor700	**CD20**	2H7	BLE
Pe-Cy7	**CD19**	J3.119	Coulter
FITC	**IgD**	Rabbit IgG	AbD
BV711	**CD21**	B-ly4	BD
PE	**CD27**	M-T271	BD
AlexaFluor647	**Bcl-6**	K112-91	BD
AlexaFluor647	**mIgG1**	MOPC21	BD
BV421	**Ki67**	B56	BD
BV421	**mIgG1**	X40	BD

Panel 3 – Follicular Helper T-cells

Fluorescent dye	*Antigen*	*clone*	*Manufact* [a]
Blue stain (UV)	**Live-Dead**	/	Invitrogen
PerCP	**CD45**	D058-1283	BD
V500	**CD3**	SP34.2	BD
Pe-Cy7	**CD4**	L200	BD
APC-H7	**CD45RA**	5H9	BD
PE	**ICOS**	C398.4A	BLE
BV711	**PD-1**	EH12.2H7	BLE
AlexaFluor647	**Bcl-6**	K112-91	BD
AlexaFluor647	**mIgG1**	MOPC21	BD
BV421	**Ki67**	B56	BD
BV421	**mIgG1**	X40	BD

Panel 4 – IFNγ-producing T-cells

Fluorescent dye	*Antigen*	*clone*	*Manufact* [a]
Blue stain (UV)	**Live-Dead**	/	Invitrogen
PerCP	**CD45**	D058-1283	BD
V500	**CD3**	SP34.2	BD
Pe-Cy7	**CD4**	L200	BD
Vioblue	**CD8**	BW135/80	MACS
APC-H7	**CD45RA**	5H9	BD
PE	**ICOS**	C398.4A	BLE
BV711	**PD-1**	EH12.2H7	BLE
AlexaFluor700	**IFNγ**	4SB3	BLE
AlexaFluor700	**mIgG1**	MOPC21	BLE

Panel 5 – Follicular Helper T-cells

Fluorescent dye	*Antigen*	*clone*	*Manufact* [a]
Blue stain (UV)	**Live-Dead**	/	Invitrogen
PerCP	**CD45**	D058-1283	BD
V500	**CD3**	SP34.2	BD
Pe-Cy7	**CD4**	L200	BD
APC-H7	**CD45RA**	5H9	BD
PE	**ICOS**	C398.4A	BLE
BV711	**PD-1**	EH12.2H7	BLE
APC	**CXCR5**	MU5UBEE	eBios
BV605	**CXCR3**	G025H7	BLE
BV605	**mIgG1**	MOPC21	BLE

(a) Manufacturer/distributor - **AbD**: AbD Serotec - Bio-Rad, Colmar, France; **BD**: BD Biosciences, Rungis, France; **BLE**: Biolegend, Ozyme, St Quentin-en-Yvelines, France**; Coulter**: Beckman Coulter, Villepinte, France**; eBios** : eBiosciences™, Paris; **Invitrogen**: Life Technologies SAS, Saint Aubin, France; **MACS**: Miltenyi Biotec –Paris, France.
