# Supplementary material for: Distinct Features of Germinal Center Reactions in Macaques Infected by SIV or Vaccinated with a T-Dependent Model Antigen
Source: Viruses. 2021 Feb 9;13(2):263. doi: 10.3390/v13020263 (PMC7916050; doi:10.3390/v13020263)
Supplement: Supplementary file 1 [file viruses-13-00263-s001.zip › viruses-1062458. suppl zip/Table S3-FCM2.pdf]

Table S3. Detection of BAFF receptors on B- and T-cells by flow cytometry

**Panel 6**				**Panel 7**			
**BAFF-R expression on B-cells**				**TACI expression on B-cells**			
Fluorescent dye	*Antigen*	*clone*	*Manufact*[a]	*Fluorescent dye*	*Antigen*	*clone*	*Manufact*
Blue stain (UV)	**Live-Dead**	/		Blue stain (UV)	**Live-Dead**	/	Invitrogen
PerCP	**CD45**	D058-1283	BD	PerCP	**CD45**	D058-1283	BD
V500	**CD3**	SP34.2	BD	V500	**CD3**	SP34.2	BD
Pe-Cy7	**CD19**	J3.119	Coulter	Pe-Cy7	**CD19**	J3.119	Coulter
AlexaFluor700	**CD20**	2H7	BLE	AlexaFluor700	**CD20**	2H7	BLE
FITC	**IgD**	Rabbit IgG	AbD	FITC	**IgD**	Rabbit IgG	AbD
BV711	**CD21**	B-ly4	BD	BV711	**CD21**	B-ly4	BD
BV421	**CD27**	M-T271	BD	BV421	**CD27**	M-T271	BD
PE	**BAFF-R**	11C1	BLE	PE	**TACI**	1A1	BLE
PE	**mIgG1**	MOPC21	BLE	PE	**Rat IgG2a**	RTK2758	BLE
AlexaFluor647	**Bcl-6**	K112-91	BD	AlexaFluor647	**Bcl-6**	K112-91	BD
AlexaFluor647	**mIgG1**	MOPC21	BD	AlexaFluor647	**mIgG1**	MOPC21	BD

**Panel 8**				**Panel 9**			
**BAFF-R expression on T-cells**				**TACI expression on T-cells**			
Fluorescent dye	*Antigen*	*clone*	*Manufact*[a]	*Fluorescent dye*	*Antigen*	*clone*	*Manufact*[a]
Blue stain (UV)	**Live-Dead**	/		Blue stain (UV)	**Live-Dead**	/	Invitrogen
PerCP	**CD45**	D058-1283	BD	PerCP	**CD45**	D058-1283	BD
V500	**CD3**	SP34.2	BD	V500	**CD3**	SP34.2	BD
Pe-Cy7	**CD4**	L200	BD	Pe-Cy7	**CD4**	L200	BD
APC-H7	**CD45RA**	5H9	BD	APC-H7	**CD45RA**	5H9	BD
APC	**CXCR5**	MU5UBEE	eBios	APC	**CXCR5**	MU5UBEE	eBios
BV711	**PD-1**	EH12.2H7	BLE	BV711	**PD-1**	EH12.2H7	BLE
PE	**BAFF-R**	11C1	BLE	PE	**TACI**	1A1	BLE
PE	**mIgG1**	MOPC21	BLE	PE	**Rat IgG2a**	RTK2758	BLE

(a) Manufacturer/distributor - **AbD**: AbD Serotec - Bio-Rad, Colmar, France; **BD**: BD Biosciences, Rungis, France; **BLE**: Biolegend, St Quentin-en-Yvelines, France**;** **Coulter**: Beckman Coulter, Villepinte, France; **eBios** :eBiosciences^{TM}, Paris ; **Invitrogen**: Life Technologies SAS, Saint Aubin, France.
