# Supplementary material for: Distinct Features of Germinal Center Reactions in Macaques Infected by SIV or Vaccinated with a T-Dependent Model Antigen
Source: Viruses. 2021 Feb 9;13(2):263. doi: 10.3390/v13020263 (PMC7916050; doi:10.3390/v13020263)
Supplement: Supplementary file 1 [file viruses-13-00263-s001.zip › viruses-1062458. suppl zip/Table S4-IHC.pdf]

Table S4. Antibodies for Immunohistochemistry

Antibodies	Clone	Species (Isotype)	Manufacturer [a]
CD20	L26	Mouse (IgG2a)	DAKO
CD3		Rabbit (IgG)	DAKO
CD4	1F6	Mouse (IgG1)	Novocastra
CD8		Rabbit (IgG)	Abcam
FoxP3	259D	Mouse (IgG1)	BLE
Active Caspase-3		Rabbit (IgG)	CST
Granzyme B	GrB7	Mouse (IgG2a)	DAKO
Ki67	B56	Mouse (IgG1)	BD
IRF4	MUM1	Mouse (IgG1)	DAKO
CD68	KP1	Mouse (IgG1)	DAKO
IgM		Rabbit ($F(ab)'_2$ IgG)	DAKO
IgG		Rabbit ($F(ab)'_2$ IgG)	DAKO
PD1		Goat (IgG)	Bio-techne

(a) Abcam: Paris, France; **BLE**: Biolegend, St Quentin-en-Yvelines, France; **BD**: BD Biosciences, Rungis, France ; **Bio-techne**, Lille, France ; **CST**: Cell Signaling Technology, Ozyme, St Quentin-en-Yvelines, France; **DAKO**: Les Ulis, France; **Novocastra**: Leica Biosystems, Nanterre, France.
