# Supplementary figures and images for: Distinct Features of Germinal Center Reactions in Macaques Infected by SIV or Vaccinated with a T-Dependent Model Antigen
Source: Viruses. 2021 Feb 9;13(2):263. doi: 10.3390/v13020263 (PMC7916050; doi:10.3390/v13020263)

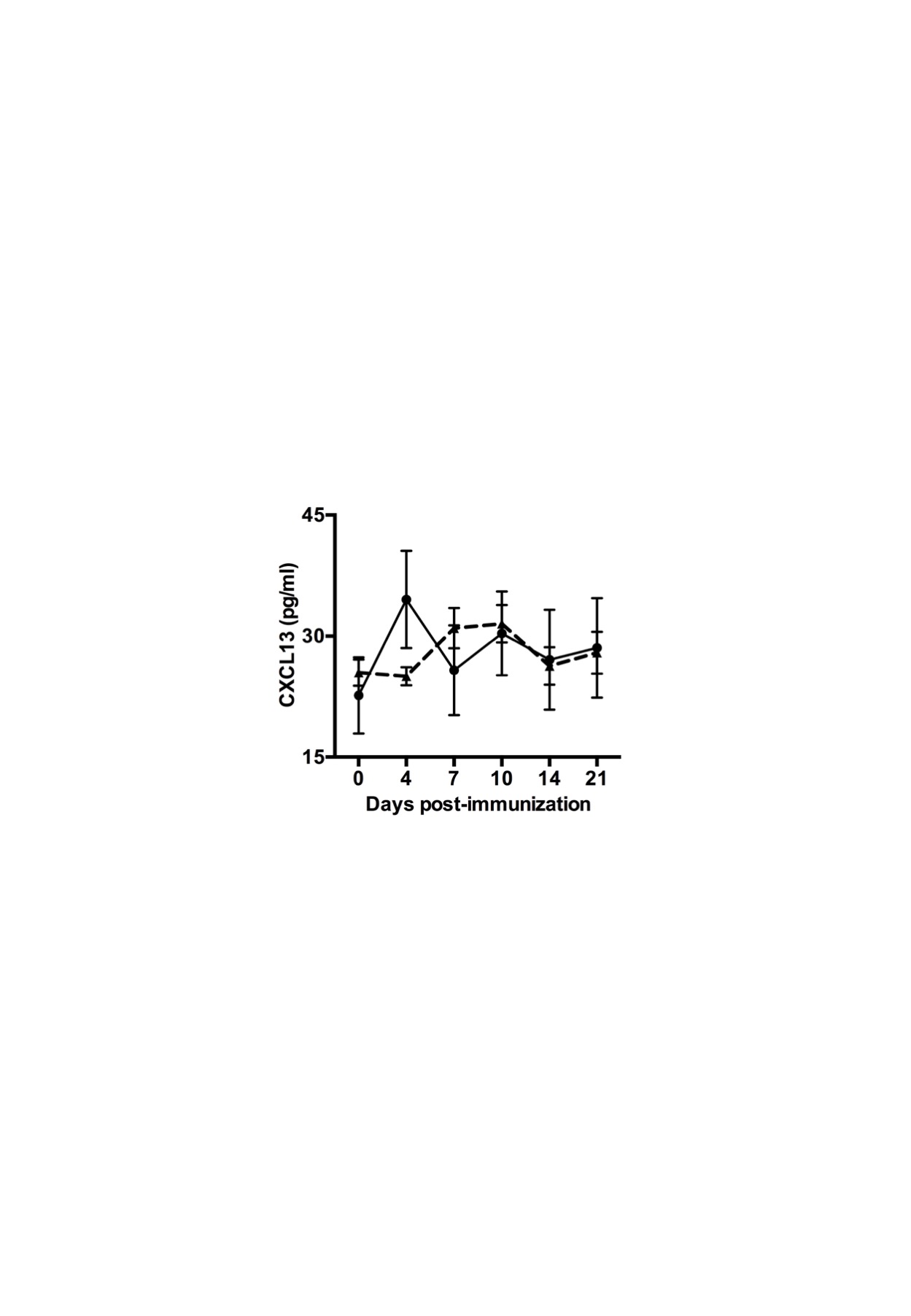

Supplement: Supplementary file 1 [file viruses-13-00263-s001.zip › viruses-1062458. suppl zip/Figure S1. Serum titers of CXCL13 during priming or boosting.jpeg]

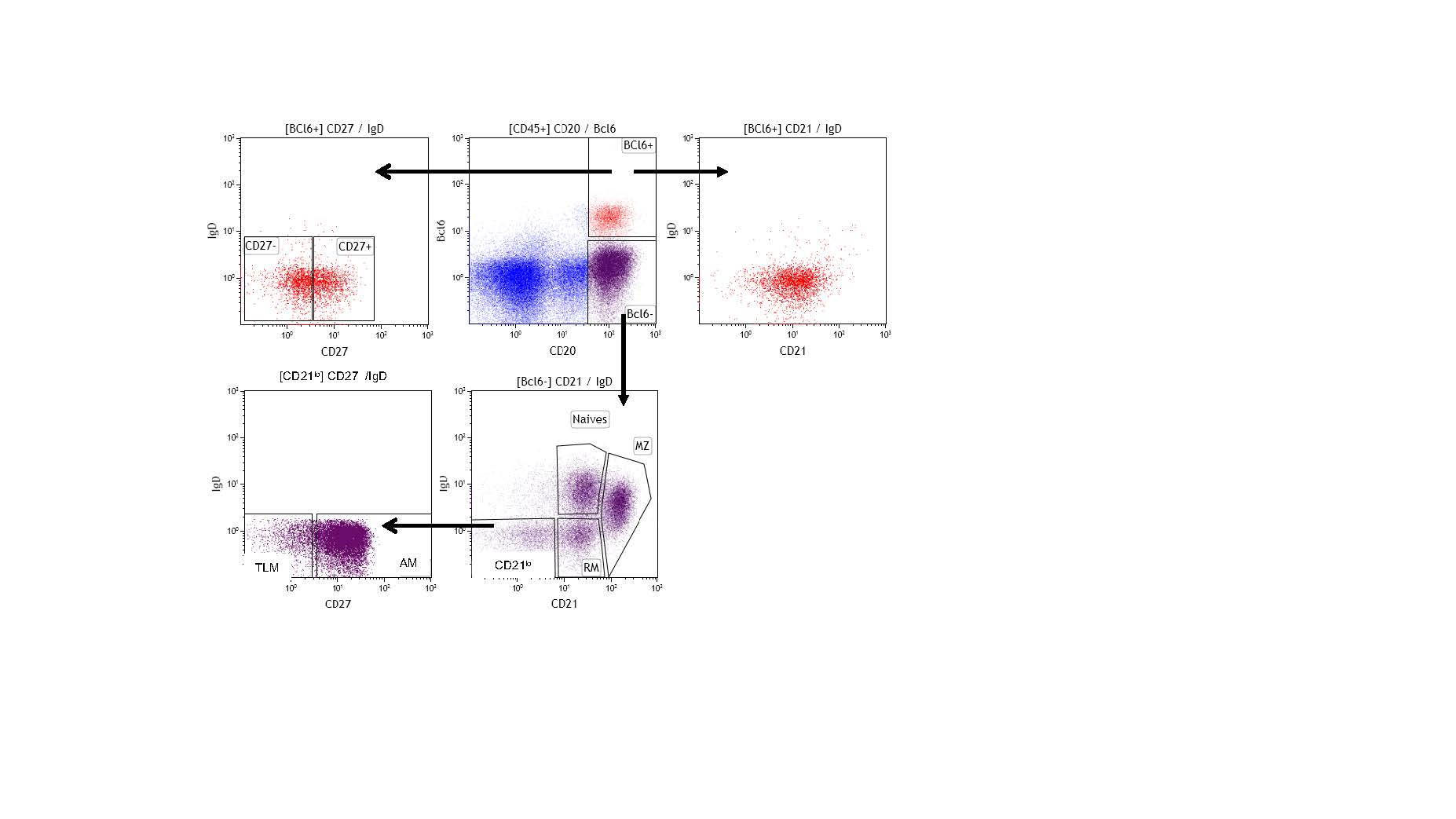

Supplement: Supplementary file 1 [file viruses-13-00263-s001.zip › viruses-1062458. suppl zip/Figure S2. B-cells in LN and spleen.jpg]

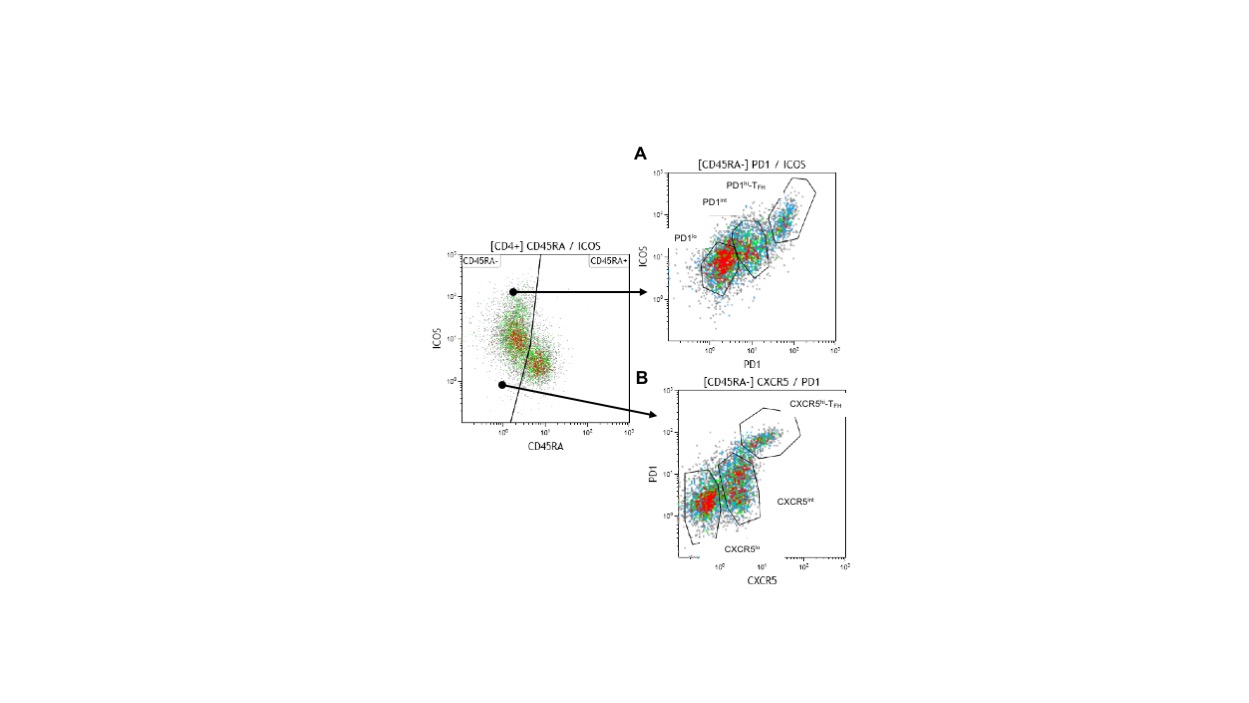

Supplement: Supplementary file 1 [file viruses-13-00263-s001.zip › viruses-1062458. suppl zip/Figure S3. mCD4 T-cell subsets.jpeg]
